# The Effect of Apigenin on Pharmacokinetics of Imatinib and Its Metabolite N-Desmethyl Imatinib in Rats

**DOI:** 10.1155/2013/789184

**Published:** 2013-11-28

**Authors:** Xian-yun Liu, Tao Xu, Wan-shu Li, Jun Luo, Pei-wu Geng, Li Wang, Meng-ming Xia, Meng-chun Chen, Lei Yu, Guo-xin Hu

**Affiliations:** ^1^The First Affiliated Hospital of Wenzhou Medical University, Wenzhou 325000, China; ^2^School of Pharmacy, Wenzhou Medical University, Wenzhou 325035, China; ^3^Ningbo Municipal Hospital of Traditional Chinese Medicine, Ningbo 315010, China

## Abstract

The purpose of this study was to determine the effect of apigenin on the pharmacokinetics of imatinib and N-desmethyl imatinib in rats. Healthy male SD rats were randomly divided into four groups: A group (the control group), B group (the long-term administration of 165 mg/kg apigenin for 15 days), C group (a single dose of 165 mg/kg apigenin), and D group (a single dose of 252 mg/kg apigenin). The serum concentrations of imatinib and N-desmethyl imatinib were measured by HPLC, and pharmacokinetic parameters were calculated using DAS 3.0 software. The parameters of AUC_(0−*t*)_, AUC_(0−*∞*)_, *T*_max_, *V*_*z*_/*F*, and CL_*z*_/*F* for imatinib in group B were different from those in group A (*P* < 0.05). Besides, MRT_(0−t)_ and MRT_(0−*∞*)_ in groups C and D differed distinctly from those in group A as well. The parameters of AUC_(0−*t*)_ and *C*_max_ for N-desmethyl imatinib in group C were significantly lower than those in group A (*P* < 0.05); however, compared with groups B and D, the magnitude of effect was modest. Those results indicated that apigenin in the short-term study inhibited the metabolism of imatinib and its metabolite N-desmethyl imatinib, while in the long-term study the metabolism could be accelerated.

## 1. Introduction 

**Figure 1:**
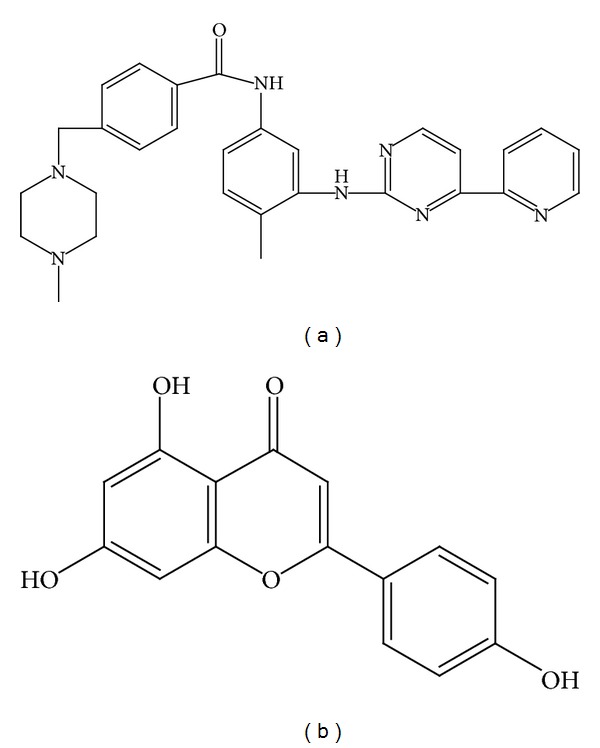
Chemical structures of imatinib (a) and apigenin (b).

Imatinib (Figure 1(a)), as we know, is a phenylamino pyrimidine derivative and a member of a new class of drugs collectively known as signal transduction inhibitors which interferes with cellular proliferation and induces apoptosis of BCR-ABL cells [1]; also imatinib interferes with the receptor tyrosine kinases for platelet-derived growth factor (PDGF), stem cell factor (SCF), and c-Kit and inhibits PDGF and SCF-mediated cellular activity in vitro [2, 3].

Imatinib mesylate is approved for the treatment of chronic myeloid leukemia (CML) in blast crisis [4], the accelerated phase of disease, or in the chronic phase of disease after not responding to interferon-*α* therapy. Studies are also being conducted to evaluate the drug in treating gastrointestinal (GI) stromal tumors (GIST, a form of sarcoma) [5], small-cell lung cancer, prostate cancer, and glioblastoma.

The major enzyme responsible for the metabolism of imatinib is CYP3A4. Other cytochrome P450 enzymes, such as CYP1A2, CYP2D6, CYP2C9, and CYP2C19, play a minor role in its metabolism. N-Demethylated piperazine derivative, which is the major circulating metabolite of imatinib, possesses in vitro activity comparable to that of imatinib [6].

Apigenin is a common flavonoid presented in a variety of plants, vegetables, fruits, and herbs, some of which are widely marketed as dietary and herbal supplements [7–11]. As an antiaggregatory, antioxidant, antibacterial, and hypertension prevention substance [12–14], the chemical structure of apigenin is showed in Figure 1(b). In addition, apigenin, to some extent, is a potent inhibitor of the cytochrome P450 (CYP) enzyme system [15–17] which is responsible for the metabolism of considerable pharmaceutical drugs.

Several important and clinically relevant interactions between flavonoids and conventional drugs have been reported over the last few years. Although various biological functions of apigenin have been demonstrated in many studies, its role in health promotion mainly depends on the intaking amount and bioavailability [9]. Given the widespread availability of apigenin, it is important to understand what effects its concomitant use may have on the disposition of medications (e.g., calcium channel blockers, antidepressants, benzodiazepines, and immunosuppressants). Specifically, induction of CYP metabolism could result in lower circulating drug concentrations, which could in turn lead to a reduction in efficacy.

Concurrent administration of other drugs or herbal products that modulate cytochrome P450 enzymes activity may alter imatinib exposure. Therefore, a combination of flavonoids and imatinib is expected. In this study, we developed a high performance liquid chromatography method for the simultaneous determination of imatinib and N-desmethyl imatinib in rat serum. The pharmacokinetics of imatinib and N-desmethyl imatinib in rats was detected after administration of apigenin.

## 2. Experimental 

### 2.1. Chemicals and Reagents

Apigenin (lot no. 520365, purity > 98.0%) was purchased from Xian Xiao Cao Botanical Development Company (Xian, China). Imatinib, N-desmethyl imatinib, and phenacetin (both purity > 98.0%) were the gifts from Rockefeller University (New York, USA). HPLC grade acetonitrile and methanol were purchased from Merck Company (Darmstadt, Germany). All other chemicals were analytical grade and used without further purification. Purified water was prepared in-house with a Milli-Q water system from Millipore (Bedford, MA, USA).

### 2.2. Equipment and Conditions

HPLC system (Agilent 1100) was equipped with quaternary pump, on-line vacuum degasser, autosampler, column compartment, diode array detector, and Agilent Chem Station Rev A.10.02. Chromatographic separation was achieved using an Agilent ZORBAX SB-C18 column (150 mm × 4.6 mm, 5 *μ*m) at 40°C at a flow rate of 1 mL/min. The concentrations of imatinib and N-desmethyl imatinib were analyzed using water-0.1% trifluoroacetic acid-acetonitrile (61 : 20 : 19, v/v/v) as the mobile phase. The detection wavelength was 282 nm, and the injection volume was 20.0 *μ*L.

### 2.3. Animals and Treatment

The Sprague-Dawley male rats (240 ± 20 g) were purchased from Shanghai CLAC Laboratory Animal Co, Ltd (Certificate no. 2007-0005). The rats were acclimatized for 7 days to laboratory conditions before initiating the experiment. Necessary approval from the Institutional Animal Ethics Committee was obtained to carry out the experiments.

### 2.4. Calibration Standards and Quality Control Samples

Stock solutions of imatinib (1 mg/mL), N-desmethyl imatinib (1 mg/mL) and IS (1 mg/mL) were separately prepared in 25 mL volumetric flasks with methanol and stored at 277 K. Working solutions for calibration and controls were prepared from the stock solution by dilution using methanol. The IS working solution (100 *μ*g/mL) was prepared by diluting its stock solution with methanol. Imatinib calibration curves were prepared using blank serum spiked at concentrations of 0.10, 0.25, 0.50, 1.00, 2.50, 5.00, 10.00, and 20.00 *μ*g/mL. N-Desmethyl imatinib calibration curves were prepared using blank serum spiked at concentrations of 0.01, 0.025, 0.05, 0.10, 0.25, 0.50, 1.00, and 2.00 *μ*g/mL.

### 2.5. Pharmacokinetic Experiment

Twenty Sprague-Dawley male rats were divided into 4 groups: A group (the control group with 0.5% CMC-Na as solvent), B group (long-term administration of 165 mg/kg apigenin for 15 days), C group (a single dose of 165 mg/kg apigenin), and D group (a single dose of 252 mg/kg apigenin). After the last administration of apigenin or 0.5% CMC-Na, the rat in each group was given a single dose of 30 mg/kg imatinib. Blood samples (500 *μ*L) were directly collected into a clean tube from the tail vein at 0 (prior to dosing), 0.5, 1, 2, 3, 4, 6, 8, 12, and 24 h after oral administration; serum was separated by centrifuging at 5000 rpm for 10 min and kept frozen at −80°C until analysis.

### 2.6. Sample Preparation

The serum samples were prepared using liquid-liquid extraction technique. A 300 *μ*L aliquot of the serum sample was taken in a 10 mL glass test tube, and on that 20 *μ*L of IS (100 *μ*g/mL) was spiked and 150 *μ*L of sodium hydroxide solution (0.01 moL/L) was added. After vortex mixing for 30 s, 3 mL of ethyl acetate was added, and the mixture was vortexed for 1 min and then centrifuged at 3000 ×g for 5 min. The organic layer was transferred into another 5 mL tube and evaporated to dryness under a stream of nitrogen gas at 40°C. The residue was reconstituted in 100 *μ*L of mobile phase and vortex-mixed for 1 min, and 20 *μ*L of aliquot was injected into the HPLC system for analysis.

### 2.7. Statistical Analysis

Experimental values were expressed as $\overset{-}{x}\pm s$. Statistical analyses of main pharmacokinetic parameters were performed by Student's unpaired test using SPSS 16.0 software. A value of *P* < 0.05 was considered to be significant between two groups.

## 3. Results 

### 3.1. Method Validation

**Figure 2:**
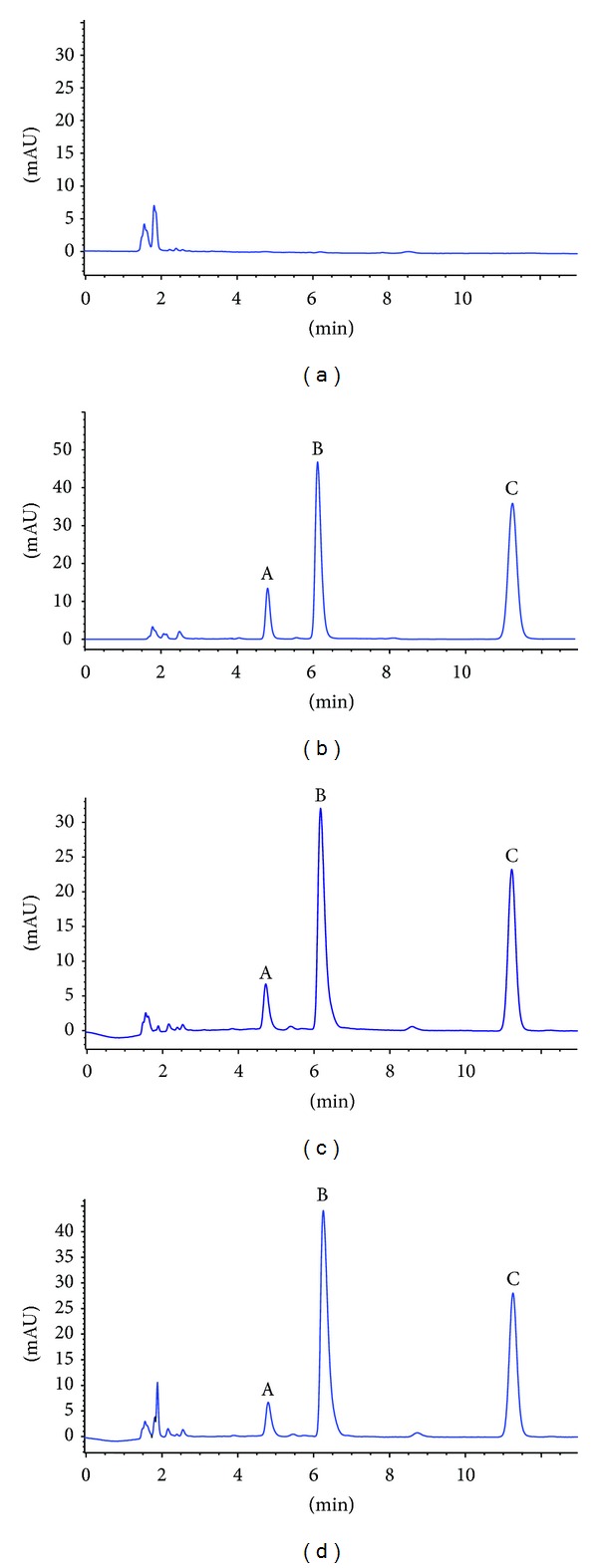
HPLC chromatograms of imatinib and N-desmethyl imatinib. (a) Representative chromatograms of blank serum. (b) Representative chromatograms of imatinib (5 *μ*g/mL) and N-desmethyl imatinib (0.5 *μ*g/mL). (c) Representative chromatograms of the serum spiked with imatinib (2.5 *μ*g/mL) and N-desmethyl imatinib (0.25 *μ*g/mL). (d) Representative chromatograms of the serum obtained at 3 h after a single oral dose of 30 mg/kg imatinib. A: N-desmethyl imatinib, B: imatinib, and C: phenacetin.

In our study, the resolution of N-desmethyl imatinib, imatinib, and internal standard was satisfactory. No interference can be observed in the HPLC chromatograms. The retention times of N-desmethyl imatinib, imatinib, and internal standard were 4.7 min, 6.2 min, and 11.4 min, respectively. The HPLC chromatograms were shown in Figure 2.

The regression equation of imatinib was *Y*_1_ = 0.1905*X*_1_ − 0.0298 (*r* = 0.9999) and its lower limit of quantitation (LLOQ) was 0.10 *μ*g/mL; the regression equation of N-desmethyl imatinib was *Y*_2_ = 0.4757*X*_2_ − 0.0088 (*r* = 0.9997) and its LLOQ was 0.01 *μ*g/mL. Both intraday and interday precisions were all less than 4.90% for imatinib and 6.26% for N-desmethyl imatinib. The method showed that the relative recoveries of imatinib and N-desmethyl imatinib were all more than 94%, whilst the absolute recoveries were all more than 76%. Blood samples at room temperature and in freezed condition showed good stability. So the method could be well applied for determinations of imatinib and N-desmethyl imatinib in rat plasma.

### 3.2. Effects of Long-Term 165 mg/kg Apigenin on Imatinib and N-Desmethyl Imatinib

**Figure 3:**
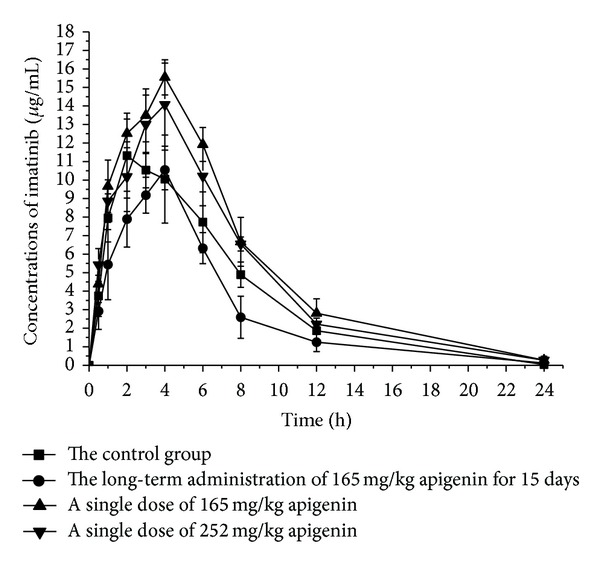
Mean concentration-time curve of imatinib in four groups (*n* = 5).

The experiment results of pharmacokinetic parameters revealed that the data of AUC_(0−*t*)_, AUC_(0−*∞*)_, *T*_max⁡_, *V*_*z*_/*F*, and CL_*z*_/*F* had a significant difference between group A and group B (*P* < 0.05) (Figure 3, Table 1). To be specific, the figures of AUC_(0−*t*)_ and AUC_(0−*∞*)_ in group B were 24.8% and 24.4% lower than those in group A, respectively, while the values of *V*_*z*_/*F* and CL_*z*_/*F* in group B increased by 100.1% and 31.3%. On the contrary, the main pharmacokinetic parameters of N-desmethyl imatinib had no great differences between the two groups.

### 3.3. Effects of Short-Term 165 mg/kg Apigenin on Imatinib and N-Desmethyl Imatinib

The main pharmacokinetic parameters of imatinib such as AUC_(0−*t*)_, AUC_(0−*∞*)_, CL_*z*_/*F*, and *C*_max⁡_ were significantly distinct between group C and group A (*P* < 0.05) (Figure 3, Table 1). Specifically, the values of AUC_(0−*t*)_ and AUC_(0−*∞*)_ in group C were 39.8% and 40.1% higher than those of group A, respectively. Concerned with *V*_*z*_/*F*, CL_*z*_/*F*, and *C*_max⁡_, pharmacokinetic parameters diminished by 10.2%, 29.3%, and 29.1%, respectively; as to *t*_(1/2)*z*_, the magnitude of effect was modest in group C.

Referred to the main pharmacokinetic parameters of imatinib, such as AUC_(0−*t*)_, AUC_(0−*∞*)_, MRT_(0−*t*)_, MRT_(0−*∞*)_, *T*_max⁡_, *V*_*z*_/*F*, CL_*z*_/*F*, and *C*_max⁡_, the results vary a lot between group B and group C (*P* < 0.05) (Figure 3, Table 1). To be specific, the figures of AUC_(0−*t*)_ and AUC_(0−*∞*)_ in group C were approximately 85% higher than the two indexes in group B. While the values of *V*_*z*_/*F* and CL_*z*_/*F* in group B both dropped a bit more decreased by 55.1% and 46.1%, respectively.

**Figure 4:**
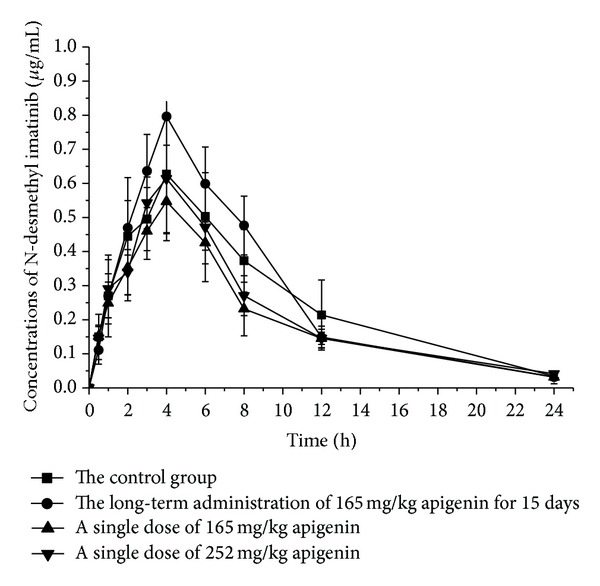
Mean concentration-time curve of N-desmethyl imatinib in four groups (*n* = 5).

In comparison with group B, the AUC_(0−*t*)_ and *C*_max⁡_ of N-desmethyl imatinib increased by 19.7% and 19.3%, respectively (Figure 4, Table 2).

### 3.4. Effects of Short-Term 252 mg/kg Apigenin on Imatinib and N-Desmethyl Imatinib

Similar to group C, the parameters of imatinib in group D revealed an enormous difference from group A and group B as well, but not to the extent of that in group C. The details were displayed in Figure 3 and Table 1.

Compared with group B, the *V*_*z*_/*F* of N-desmethyl imatinib demonstrated a decrease by 62.10%, and *C*_max⁡_ which reduced by 19.82% had a similar downward trend with *V*_*z*_/*F* (Figure 4 and Table 2).

## 4. Discussion 

The studies mainly focused on the pharmacokinetic characteristics of imatinib and its metabolite N-desmethyl imatinib, then fitting their pharmacokinetic parameters. Two-compartment model was found to meet the concentration-time curves of imatinib and N-desmethyl imatinib in four groups.

The influence of 252 mg/kg short-time apigenin group in imatinib was less than that of 165 mg/kg apigenin group, which indicated that the effect was not dose dependent. This was in good agreement with the findings of the previously published studies [18]. The authors suggested that the main part of the ingested apigenin was either excreted unabsorbed or was rapidly metabolized after absorption. Besides, the imatinib in the long-term group and short-term groups had significant differences related to AUC_(0−*t*)_, AUC_(0−*∞*)_, MRT_(0−*t*)_, MRT_(0−*∞*)_, *V*_*z*_/*F*, CL_*z*_/*F*, and *C*_max⁡_ (*P* < 0.05). As a result, the metabolism of imatinib was inhibited by a single dose of application in vivo, while it was accelerated by a long-term oral administration of apigenin.

Natural flavonoids, such as flavone, tangeretin, and nobiletin, could affect the human CYP activities when administered orally [19]. CYP3A exhibited large interindividual variation [20] and represented the largest piece of CYP enzymes expressed in the human liver [21]. Many drug-interaction between herbal medicines and anticarcinogen may contribute to the inhibitory effect on CYP3A4 [22, 23].

Imatinib has very good oral absorption, whose oral bioavailability is close to 97% [24]. It had wide tissue distribution as well, which meant that a small change in the binding protein proportion would cause large variances in pharmacology. Imatinib is not only the substrate of CYP3A4, but also the inhibitor of CYP3A4. The plasma concentrations of CYP3A4 substrate, such as simvastatin and pimozide, may be elevated by imatinib [17]. Imatinib could not only affect CYP3A4 but also influence CYP2D6, CYP2C9, and CYP2C19 in various degrees [25–27]. The results got from the research supported that imatinib exposure was related to hematologic response; thus an increase in imatinib exposure produced by apigenin is likely to be clinically relevant and may lead to adverse drug reaction.

From the analysis, the results also indicated that a single dose administration could reduce the metabolism rate of imatinib, increase bioavailability, and extend the resistance time of prototype drug in vivo. Those changes might be related to a potential interaction between apigenin and CYP enzymes.

Given the widespread availability of apigenin, it was important to understand what effects its concomitant use might have on the disposition of imatinib; therefore, the research was carried out as expected.

## 5. Conclusion 

In summary, patients taking imatinib should pay attention to the intake of the food with apigenin. The inhibitory effects of single-dose apigenin would result in higher concentrations of imatinib in the short term but lower in the long term exposure. Attention has to be paid on the dose adjustments in order to reduce the adverse reaction of drug interactions when apigenin and imatinib are administrated concurrently. Up to now, there are few reports about the effects of traditional Chinese medicine and food composition on imatinib metabolism. All above results showed preponderance of the evidence for clinical rational use of imatinib, and provided a new perspective for the understanding of apigenin.

## Figures and Tables

**Table 1:** The main pharmacokinetic parameters of imatinib in four groups (*n* = 5).

Parameters	A	B	C	D
AUC_(0–*t*)_ (mg·h·L^−1^)	90.618 ± 11.34	68.158 ± 7.279*	126.678 ± 12.499^∗,∗∗^	113.095 ± 8.918^∗,∗∗^
AUC_(0–*∞*)_ (mg·h·L^−1^)	90.988 ± 11.585	68.805 ± 7.432*	127.514 ± 12.332^∗,∗∗^	114.502 ± 8.634^∗,∗∗^
MRT_(0–*t*)_ (h)	5.718 ± 0.274	5.479 ± 0.512	6.121 ± 0.33**	6.049 ± 0.351**
MRT_(0–*∞*)_ (h)	5.804 ± 0.376	5.707 ± 0.579	6.274 ± 0.337**	6.337 ± 0.532**
*t*_(1/2)*z*_ (h)	2.304 ± 0.954	3.423 ± 1.361	2.82 ± 1.069	3.456 ± 0.418
*T*_max⁡_ (h)	3.0 ± 1.003	2.0 ± 0.004*	3.8 ± 0.447**	3.6 ± 0.548**
V_*z*_/*F* (kg·L^−1^)	1.081 ± 0.372	2.163 ± 0.875*	0.971 ± 0.396**	1.315 ± 0.209**
CL_z_/F (L·min^−1^·kg^−1^)	0.335 ± 0.051	0.44 ± 0.05*	0.237 ± 0.023^∗,∗∗^	0.263 ± 0.019^∗,∗∗^
*C*_max⁡_ (mg·L^−1^)	12.074 ± 2.023	10.544 ± 0.931	15.583 ± 0.86^∗,∗∗^	14.388 ± 1.753^∗,∗∗^

*P < 0.05 in comparison to group A; **P < 0.05 in comparison to group B.

**Table 2:** The main pharmacokinetic parameters of N-desmethyl imatinib in four groups (*n* = 5).

Parameters	A	B	C	D
AUC_(0–*t*)_ (mg·h·L^−1^)	5.977 ± 1.626	6.335 ± 1.105	4.797 ± 0.602**	5.262 ± 0.89
AUC_(0–*∞*)_ (mg·h·L^−1^)	6.827 ± 2.595	6.672 ± 0.797	5.05 ± 0.797	5.545 ± 0.814
MRT_(0–*t*)_ (h)	7.085 ± 1.135	6.573 ± 0.544	7.227 ± 0.525	7.326 ± 0.53
MRT_(0–*∞*)_ (h)	9.718 ± 4.636	7.504 ± 0.189	8.397 ± 1.545	8.679 ± 1.301
*t*_(1/2)*z*_ (h)	5.495 ± 3.92	4.083 ± 0.436	4.906 ± 2.093	5.376 ± 1.495
*T*_max⁡_ (h)	3.6 ± 0.894	4.0 ± 0.003	4.0 ± 0.005	3.8 ± 0.447
V_*z*_/*F* (kg·L^−1^)	32.802 ± 10.255	26.843 ± 4.486	40.738 ± 12.777	43.512 ± 16.116**
CL_z_/F (L·min^−1^·kg^−1^)	4.913 ± 1.782	4.552 ± 0.592	6.066 ± 0.992	5.507 ± 0.834
*C*_max⁡_ (mg·L^−1^)	0.678 ± 0.147	0.797 ± 0.073	0.547 ± 0.091**	0.639 ± 0.13**

*P < 0.05 in comparison to group A; **P < 0.05 in comparison to group B.

## References

[1] Wu J, Meng F, Lu H (2008). Lyn regulates BCR-ABL and Gab2 tyrosine phosphorylation and c-Cbl protein stability in imatinib-resistant chronic myelogenous leukemia cells. *Blood*.

[2] Kris MG, Natale RB, Herbst RS (2003). Efficacy of gefitinib, an inhibitor of the epidermal growth factor receptor tyrosine kinase, in symptomatic patients with non-small cell lung cancer: a randomized trial. *The Journal of the American Medical Association*.

[3] Litz J, Krystal GW (2006). Imatinib inhibits c-Kit-induced hypoxia-inducible factor-1*α* activity and vascular endothelial growth factor expression in small cell lung cancer cells. *Molecular Cancer Therapeutics*.

[4] Talpaz M, Shah NP, Kantarjian H (2006). Dasatinib in imatinib-resistant Philadelphia chromosome-positive leukemias. *The New England Journal of Medicine*.

[5] Demetri GD, von Mehren M, Blanke CD (2002). Efficacy and safety of imatinib mesylate in advanced gastrointestinal stromal tumors. *The New England Journal of Medicine*.

[6] Burger H, Nooter K (2004). Pharmacokinetic resistance to imatinib mesylate: role of the ABC drug pumps ABCG2 (BCRP) and ABCB1 (MDR1) in the oral bioavailability of imatinib. *Cell Cycle*.

[7] Beara IN, Lesjak MM, Jovin ED (2009). Plantain (*Plantago* L.) species as novel sources of flavonoid antioxidants. *Journal of Agricultural and Food Chemistry*.

[8] Li C, Du H, Wang L (2009). Flavonoid composition and antioxidant activity of tree peony (*Paeonia* section *Moutan*) yellow flowers. *Journal of Agricultural and Food Chemistry*.

[9] Lu XY, Sun DL, Chen ZJ (2010). Relative contribution of small and large intestine to deglycosylation and absorption of flavonoids from *Chrysanthemun morifolium* extract. *Journal of Agricultural and Food Chemistry*.

[10] Panoino G, Courts FL, Lombardo S, Mauromicale G, Williamson G (2010). Caffeoylquinic acids and flavonoids in the immature Inflorescence of globe artichoke, wild cardoon, and cultivated cardoon. *Journal of Agricultural and Food Chemistry*.

[11] Qiu Y, Liu Q, Beta T (2009). Antioxidant activity of commercial wild rice and identification of flavonoid compounds in active fractions. *Journal of Agricultural and Food Chemistry*.

[12] Matsumoto S, Kimura S, Segawa H (2005). Efficacy of the third-generation bisphosphonate, zoledronic acid alone and combined with anti-cancer agents against small cell lung cancer cell lines. *Lung Cancer*.

[13] Tarumoto T, Nagai T, Ohmine K (2004). Ascorbic acid restores sensitivity to imatinib via suppression of Nrf2-dependent gene expression in the imatinib-resistant cell line. *Experimental Hematology*.

[14] Du W, Sun C, Liang Z, Han Y, Yu J (2012). Antibacterial activity of hypocrellin A against *Staphylococcus aureus*. *World Journal of Microbiology and Biotechnology*.

[15] Lampropoulos P, Lambropoulou M, Papalois A (2013). The role of apigenin in an experimental model of acute pancreatitis. *Journal of Surgical Research*.

[16] Shimada H, Eto M, Ohtaguro M (2010). Differential mechanisms for the inhibition of human cytochrome P450 1A2 by apigenin and genistein. *Journal of Biochemical and Molecular Toxicology*.

[17] Ho PC, Saville DJ, Wanwimolruk S (2001). Inhibition of human CYP3A4 activity by grapefruit flavonoids, furanocoumarins and related compounds. *Journal of Pharmacy and Pharmaceutical Sciences*.

[18] Hellen M, Adrian B, Guenther W (2006). Bioavailability of apigenin from apiin-rich parsley in humans. *Annals of Nutrition and Metabolism*.

[19] Hodek P, Trefil P, Stiborová M (2002). Flavonoids-potent and versatile biologically active compounds interacting with cytochromes P450. *Chemico-Biological Interactions*.

[20] Hunt CM, Westerkam WR, Stave GM (1992). Effect of age and gender on the activity of human hepatic CYP3A. *Biochemical Pharmacology*.

[21] Li J, Karlsson MO, Brahmer J (2006). CYP3A phenotyping approach to predict systemic exposure to EGFR tyrosine kinase inhibitors. *Journal of the National Cancer Institute*.

[22] Yang CS, Lambert JD, Sang S (2009). Antioxidative and anti-carcinogenic activities of tea polyphenols. *Archives of Toxicology*.

[23] Surh YJ, Ferguson LR (2003). Dietary and medicinal antimutagens and anticarcinogens: molecular mechanisms and chemopreventive potential—highlights of a symposium. *Mutation Research*.

[24] Panigrahi I, Naithani R (2006). Imatinib mesylate: a designer drug. *Journal of Association of Physicians of India*.

[25] Wang Y, Zhou L, Dutreix C (2008). Effects of imatinib (Glivec) on the pharmacokinetics of metoprolol, a CYP2D6 substrate, in Chinese patients with chronic myelogenous leukaemia. *British Journal of Clinical Pharmacology*.

[26] Yamakawa Y, Hamada A, Nakashima R (2011). Association of genetic polymorphisms in the influx transporter SLCO1B3 and the efflux transporter ABCB1 with imatinib pharmacokinetics in patients with chronic myeloid leukemia. *Therapeutic Drug Monitoring*.

[27] Filppula AM, Neuvonen M, Laitila J, Neuvonen PJ, Backman  JT (2013). Autoinhibition of CYP3A4 leads to important role of CYP2C8 in imatinib metabolism: variability in CYP2C8 activity may alter plasma concentrations and response. *Drug Metabolism and Disposition*.

